# The Southern Surgical and Gynecological Association

**Published:** 1888-12

**Authors:** 


### Society Notes.

The Southern Surgical and Gynecological Association held its annual meeting at Birmingham, Alabama, on December 4, 5 and 6. The meeting was called to order by the President, Prof. W. H. Haggard, of Nashville, Tennessee. The attendance was above 100, consisting of the leading surgeons of the South. At the opening of the first session, a motion was passed, declining with thanks, several excursions tendered the| association.—They declared themselves unanimously for business only.

The Judicial Council reported as follows, on the qualifications for membership: Every applicant for membership must be recommended by two members of the Association. He must present to the Judicial Council, a paper of his own composition, on some surgical or gynecological subject. The merits of this paper to decide the question of his admission.

No paper shall be read before this Association except it be upon some surgical or gyneological topic, and all such papers must receive the endorsement of the Committee of Publication, before they appear in print, as having been read before this Association. This report was thorougly discussed, and unanimously adopted. The sentiment was for a "high standard."

The only invitation to recreative enjoyment accepted by the Association, was a musical one, tendered by the Mendelssohn Club, which proved quite enjoyable to the lovers of fine music. The annual orator of the occasion, Dr. W. F. Hyer, made his appearance on the stage at the middle of the concert, and delivered quite a "taking" address—was loudly applauded.

The first paper read was by Prof. W. B. Rogers, of Memphis, Tennessee. Subject, Gastrostomy. In conjunction with this subject a case was reported, and the patient presented to the Association. Professor Rogers operated in May, 1888; subject: male; white; 20 years of age. Operation performed on account of cicatricial stricture of the œsophagus, due to swallowing concentrated lye in June, 1887. Finger's incision was followed; stomach secured to abdominal walls by hare-lip pins and silk sutures; gastric opening made on 10th day; patient in meantime supported by rectal enemata and milk per os. Chloroform, the anæsthetic; time of operation, 1 hour and 10 minutes. Rapid recovery, with slight improvement in deglutition. Health and strength rapidly regained.

Prof. W. H. Wathen, of Louisville, Kentucky, presented uterus with ovaries and tubes removed for carcinoma, also displayed forceps with which he clamped the vessels of broad ligament, and controlled all hemorrhage. Neither ligatures nor sutures were used. Time of operation, 20 minutes. Patient made rapid recovery. The case was very clearly and concisely reported, and the subject beautifully handled.*

Prof. Virgil O. Hardon, of Atlanta, Georgia, read "the paper" of the occasion; taking a mean ground between the two theories, one of the existence of such a thing as chronic pelvic cellulitis, and the other, that no such thing exists. His classification of pelvic indurations was clear, and his arguments conclusive.

Dr. J. M. Taylor, of Corinth, Miss., read a paper on antiseptics. He is decidedly skeptical of their value, and contends for cleanliness versus antiseptics. His views raised much discussion, for the advocates of strict antiseptic methods were plentiful.

Dr. Bedford Brown, of Virginia, reported quite a list of cases of uterine fibroids, treated by ergot and hypophosphites. His treatment is quiet encouraging to those inclined to the medical treatment of these growths.

Dr. W. F. Westmoreland, jr., of Atlanta, Ga., described the application of Plaster of Paris, as a splint in fractures of the lower extremity, especially of the female. He treats the limb in the extended position, carrying his rollers from toe to crest of ilium.

Dr. John Brownrigg, of Columbus, Miss., presented splints of his own device for treatment of fractures of the forearm. He keeps up constant extension of the wrist in Colles' fracture, and at the same time allows slight movement all the time, at that joint. His success is most gratifying.

Prof. J. H. Blanks,† of Nashville, Tenn., [?] reported some cases of lacerated perineum, with unique symptoms, and the subject elicited much discussion.

"Shock of Injury, and its Effects," was most admirably pictured and handled by Dr. John R. Page, of Birmingham, Ala. The Doctor endorses the practice of delay, in operative procedures, in cases of railroad injury—secondary amputations.

Dr. W. D. Bizzell, of Atlanta, Ga., presented a paper, "Certain Forms of Menorrhagia," which was most exhaustive. He advocates tamponing the cavity of the uterus, reporting beautiful results.

"Operative procedures in Hypertrophy of the Prostate," by Dr. R. D. Webb, of Birmingham, Ala., was one of the most classical and exhaustive papers read.

Space forbids the mentioning of numerous other papers read. Your correspondent having been compelled to leave before the meeting was over, will be pardoned for merely mentioning the authors of other exceedingly interesting papers read during the first two days.

Dr. J. D. S. Davis, of Alabama, "Electrolysis in Morbid Alterations produced in prostate by Gonorrhœa of the Urethra." "Dermoid Cysts of Coccygeal Region," by E. J. Beall, M. D., of Texas.* "Surgical Tuberculosis," by Dr. W. L. Chew, of Alabama.

The following officers were selected for the ensuing year:

Hunter McGuire, M. D., Va., president.

W. O. Roberts, M. D., Ky., vice president.

Bedford Brown, M. D., Va., vice president.

The next meeting will be held in Nashville, Tenn., on the second Tuesday in December, 1889. W. B. R.

---

*[These papers appear in this issue of THE JOURNAL.—Ed].

† [We believe our correspondent has reference to our friend Dr. J. H. Blanks, of Meridian, Miss.—Ed.]

